# Functional and structural characterization of plastidic starch phosphorylase during barley endosperm development

**DOI:** 10.1371/journal.pone.0175488

**Published:** 2017-04-13

**Authors:** Jose A. Cuesta-Seijo, Christian Ruzanski, Katarzyna Krucewicz, Sebastian Meier, Per Hägglund, Birte Svensson, Monica M. Palcic

**Affiliations:** 1 Carlsberg Research Laboratory, J.C. Jacobsens Gade 4, DK-1799 Copenhagen V, Denmark; 2 Novo Nordisk A/S, Måløv, Denmark; 3 DTU, Department of Chemistry, Technical University of Denmark, Kemitorvet, Building 207, DK-2800 Kgs. Lyngby, Denmark; 4 DTU Department of Biotechnology and Biomedicine, Proteomics Core, Technical University of Denmark, Matematiktorvet, Building 301, DK-2800 Kgs. Lyngby, Denmark; 5 DTU Department of Biotechnology and Biomedicine, Enzyme and Protein Chemistry, Technical University of Denmark, Elektrovej Building 375, DK-2800 Kgs. Lyngby, Denmark; 6 Department of Biochemistry and Microbiology, University of Victoria, Victoria, British Columbia, Canada; Virginia Polytechnic Institute and State University, UNITED STATES

## Abstract

The production of starch is essential for human nutrition and represents a major metabolic flux in the biosphere. The biosynthesis of starch in storage organs like barley endosperm operates via two main pathways using different substrates: starch synthases use ADP-glucose to produce amylose and amylopectin, the two major components of starch, whereas starch phosphorylase (Pho1) uses glucose-1-phosphate (G1P), a precursor for ADP-glucose production, to produce α-1,4 glucans. The significance of the Pho1 pathway in starch biosynthesis has remained unclear. To elucidate the importance of barley Pho1 (*Hv*Pho1) for starch biosynthesis in barley endosperm, we analyzed *Hv*Pho1 protein production and enzyme activity levels throughout barley endosperm development and characterized structure-function relationships of *Hv*Pho1. The molecular mechanisms underlying the initiation of starch granule biosynthesis, that is, the enzymes and substrates involved in the initial transition from simple sugars to polysaccharides, remain unclear. We found that *Hv*Pho1 is present as an active protein at the onset of barley endosperm development. Notably, purified recombinant protein can catalyze the *de novo* production of α-1,4-glucans using *Hv*Pho1 from G1P as the sole substrate. The structural properties of *Hv*Pho1 provide insights into the low affinity of *Hv*Pho1 for large polysaccharides like starch or amylopectin. Our results suggest that *Hv*Pho1 may play a role during the initiation of starch biosynthesis in barley.

## Introduction

Starch is the most important storage carbohydrate of higher plants and plays important roles during their life cycle. Our economy depends on starch as primary food source, to exploit its unique physicochemical properties in numerous industrial applications, as feedstock and for the production of bioethanol [1]. Crop plants store large amounts of starch in the amyloplasts of highly specialized storage compartments like potato tubers or barley endosperm. In the amyloplasts, starch accumulates in the form of starch granules, which are primarily composed of two homopolymers of glucose: branched amylopectin and linear amylose.

The enzymes responsible for the biosynthesis of starch granules were amongst the first enzymes studied. An initial hint as to how starch is synthesized was already given by Hanes, Green and Stumpf in the early 1940s. They reported on a “series of reversible chemical reactions by which glucose is transformed” [2,3] through “phosphorolytic activity”. It was soon proposed that the newly discovered starch phosphorylase (in combination with branching enzyme) must be a key enzyme in the biosynthesis of starch in plants [4]. Subsequent discoveries of the ADP-glucose utilizing starch synthases (SS) from the retaining GT5 enzyme family, however, shifted the main scientific focus and primary research efforts on starch biosynthesis. In the current model, the biosynthesis of starch in plants is described to primarily proceed via granule bound starch synthase (GBSS) and soluble starch synthases (SSI, SSII, SSIII and SSIV) [5].

Recent biochemical and genetic studies in crop plants renew the hypothesis that starch phosphorylase plays a key role in the production and initiation of storage starch. *In vitro* experiments with potato tubers showed high levels of glucose incorporation into tuber starch using [^14^C] G1P as a glucosyl donor [6]. Rice mutants lacking plastidial starch phosphorylase have a shrunken endosperm phenotype and altered starch structures [7]. Enzymatic analyses of rice starch phosphorylase furthermore show that *Os*Pho1 favors the synthetic reaction route over the phosphorolytic even with G1P / Pi ratios that were previously thought to favor the latter [8], which is in line with previous studies in *C*. *reinhardtii* [9]. It was furthermore shown that *Os*Pho1 forms a functional protein complex with branching enzymes of rice *in vitro* [10], which was able to synthesize amylopectin *de novo* using G1P as the sole substrate without addition of an acceptor. In addition, *Hv*Pho1 transcripts are already present at the early onset of endosperm development [11]. These data strongly suggest that starch phosphorylase is a key enzyme during starch biosynthesis. However, transcripts are poor predictors of protein production and enzymatic activity [12,13].

Quantitative data about starch phosphorylase protein abundance and enzyme activity are lacking and no crystal structure of a plastidic plant starch phosphorylase has been reported so far. It is known that starch phosphorylases are members of the CAZy GT35 family of glycosyltransferases [14] and catalyze the reversible transfer of glucosyl residues from a glucose donor onto the non-reducing end of an α-glucan chain [3]. The minimal acceptor length of α-1,4-glucan phosphorylases is maltotriose [14]. Plants contain at least two different α-1,4-glucan phosphorylase isozymes (Pho1 and Pho2) located in different cellular compartments [15]. While Pho2 is localized in the cytosolic compartment, Pho1 is a true starch phosphorylase that acts on starch in the plastids of the plant cell [7,16]. Pho1 contains a specific insertion that is not found in Pho2 enzymes. This 78 amino acid residues long insertion was named L78 in sweet potato *Ib*Pho1 based on its amino acid content. It was suggested that L78 forms a flexible loop in Pho1 enzymes [17,18]. However, the exact function of this insertion has not been elucidated.

To shed new light on the role of *Hv*Pho1 during starch biosynthesis in barley endosperm, we carried out a comprehensive functional and structural characterization of *Hv*Pho1. Protein production and functional enzyme assays throughout endosperm development show the importance of *HvPho1* for starch biosynthesis. Active *Hv*Pho1 was already found present at the initial stage of endosperm development, while the analysis of *Hv*Pho1 kinetics showed that highly purified recombinant *Hv*Pho1 produced α-1,4 glucans *de novo*. We also determined three crystal structures of *Hv*Pho1 to obtain insights into the structural basis for its function and acceptor binding as well as its low affinity for complex polysaccharides such as starch and amylopectin. Taken together, these results provide essential information about starch synthesis initiation in crop plants like barley and shed new light on the functionality of Pho1 in starch biosynthesis.

## Results

### Immunological and zymographic detection of *Hv*Pho1 throughout barley endosperm development

**Fig 1:**
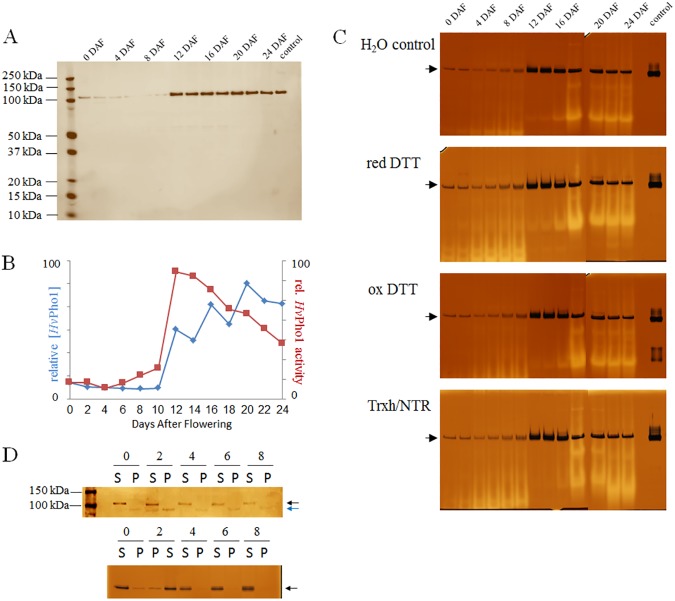
Abundance levels and enzymatic activity of *Hv*Pho1 during barley endosperm development. (A) *Hv*Pho1 protein abundance from barley endosperm extracts analyzed via immunoblot at 2 d intervals. Only one band is visible just above 100 kDa in accordance with an expected mass of 105 kDa. (B) Relative quantification of the data from panel A (blue line) and activity from panel C (H_2_O control; red line). (C) Starch phosphorylase activity probed in 2 day intervals as for panel A but with native gels and Lugol coloring of activity products. Strong synthetic activity appears as a dark stained band and is marked with a black arrow. White bands and smears represent amylolytic activities. All gels include a recombinant *Hv*Pho1 control as the right-most band. The different redox treatments are indicated next to each gel. (D) Immunoblot (top) and native gel (bottom) analysis of *Hv*Pho1 protein abundance on buffer soluble (S) protein and buffer insoluble (P) protein fractions of barley endosperm between 0 and 8 DAF. Numbers indicate the DAF. Arrows mark the position of the two relevant bands in the immunoblot and the position of the (single) activity band in the zymogram.

The amount of *Hv*Pho1 throughout endosperm development between 0 and 24 days after flowering (DAF) was determined using semi-quantitative immunological analysis with highly pure recombinant *Hv*Pho1 as a standard (Fig 1A). Our results show that *Hv*Pho1 is already present at 0 DAF with its abundance decreasing in the following 10 days. This is followed by a large increase in protein production at 12 DAF. Protein levels of *Hv*Pho1 remain high until 24 DAF. Native gel analysis of the same protein samples shows that *Hv*Pho1 activity does not completely correlate with *Hv*Pho1 abundance (Fig 1B and 1C). The activity of *Hv*Pho1 first decreased and subsequently increased between 0 DAF and 10 DAF. The activity levels then greatly increased at 12 DAF but steadily decreased thereafter.

The discrepancy between protein abundance and activity levels could be due to specific modifications of *Hv*Pho1 during endosperm development. We tested the hypothesis that the redox state could be influencing the activity levels of *Hv*Pho1 by incubating soluble barley endosperm protein extracts harvested at 0 to 24 DAF with either H_2_O (as control), reduced DTT, oxidized DTT or a barley thioredoxin system composed of highly purified recombinant thioredoxin (*Hv*Trxh2), thioredoxin reductase (*Hv*NTR2) and NADPH [19], prior to native gel analysis. No significant effect on *Hv*Pho1 activity was observed (Fig 1C). The activity of purified recombinant *Hv*Pho1 (control lane) was also not affected by the same treatment. In contrast, the overall amylolytic activities increased when plant protein samples were treated with reduced DTT or the thioredoxin system.

These analyses were done using soluble grain extracts. Interestingly a protein band specific for the anti-*Hv*Pho1 antibody, of approximately 20 kDa less than the full-length *Hv*Pho1, partially associates with the insoluble protein fraction during the onset of endosperm development (0 DAF to 6 DAF). The fragment was most prominent at 2 DAF (Fig 1D), although still representing only a minor fraction of total Pho1 abundance. Native gel analysis on those fractions showed that both proteins (full-length and full-length minus ~20 kDa) are active with glycogen and G1P as substrates (Fig 1D).

### Assessment of the oligomeric state of active *Hv*Pho1 and effect of the L78 insertion of Pho1

**Fig 2:**
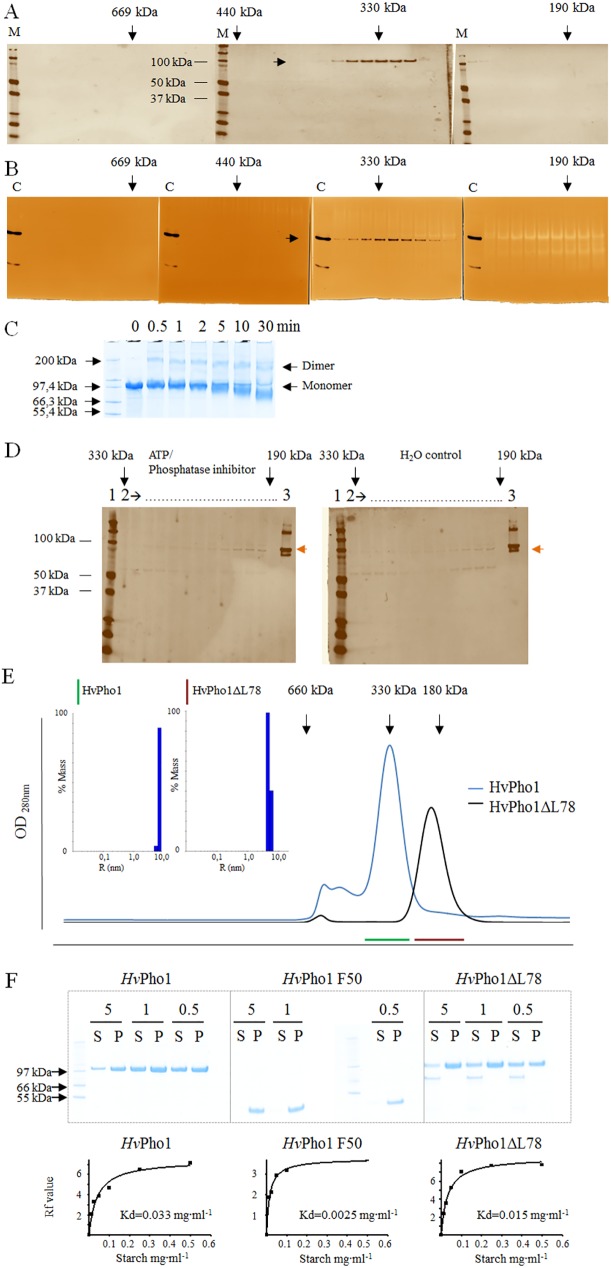
Apparent molecular weights and affinity of *Hv*Pho1 constructs. (A), (B), (C) Total barley endosperm soluble protein loaded onto a 26/60 Superdex S-200 SEC column. (A) Protein fractions as analyzed by SDS-PAGE/immunoblot using anti-*Hv*Pho1 polyclonal antibodies and (B) Native gels using glycogen as in gel glycosyl acceptor and G1P as glycosyl donor. The first lane (marked M) on each immunoblot is protein molecular weight ladder. The first lane in each native gel (marked C) is the recombinant *Hv*Pho1 control. Arrows on top of the gels indicate molecular weight of the protein fraction according to column calibration. (C) Chemical cross-linking of *Hv*Pho1 dimers in solution. *Hv*Pho1 was incubated for the indicated times with 0,15% (v/v) glutaraldehyde. Formation of *Hv*Pho1 cross-linked dimers is indicated. (D) Protein fractions as analyzed by SDS-PAGE/immunoblot using anti-*Hv*BeIIb polyclonal antibodies. Total barley endosperm protein was either incubated either with (left gel) or without (right gel) 1 mM ATP and 2.5 mM protein phosphatase inhibitor cocktail prior to size separation via SEC. Lane 1: protein molecular weight ladder, lanes marked 2: protein fractions from SEC ranging between 330 kDa and 190 kDa; lane 3: recombinant *Hv*BeIIb purified from *E*. *coli*. Brown arrows to the right of both gels indicate HvBeIIb. (E) SEC profile of *Hv*Pho1 (blue) and *Hv*Pho1ΔL78 (black). The inserts are DLS analyses of the hydrodynamic sizes of the two proteins. The green and brown lines represent the SEC peak fractions of the respective proteins used for SEC analysis. Arrows on top indicate molecular weight according to column calibration. (F) The affinity of recombinant *Hv*Pho1, *Hv*Pho1F50 and *Hv*Pho1ΔL78 for amylopectin and starch assessed by analysis of starch bound and unbound protein *in vitro* in two ways: Top: By incubation of the proteins with 5, 1 and 0.5 mg∙ml^-1^ amylopectin and successive analysis of soluble (S) and pellet–amylopectin bound (P) fraction with SDS-PAGE. The concentrations of amylopectin are indicated over each S/P pair. Bottom: analysis via native gel with in gel starch as an interaction partner. Rf values are plotted versus the starch concentration in the gels. The resultant affinity constants for half maximum binding are given.

Data about the oligomeric state of *Hv*Pho1 *in vitro* and *in vivo* were lacking and we assessed the hydrodynamic properties of the enzyme prior to structural studies. Therefore, we used size exclusion chromatography (SEC), native gel electrophoresis and dynamic light scattering (DLS) analysis to assess the oligomeric state of *Hv*Pho1 in barley endosperm. The SEC elution pattern of *Hv*Pho1 ranged from 300 kDa to 360 kDa with a maximum at 330 kDa (Fig 2A and 2B). The SDS-PAGE gel migration pattern of those bands corresponds to a size of just above 100 kDa in good agreement with the calculated monomeric size of *Hv*Pho1 of 105 kDa.

To determine if the protein forms trimers in solution, we chemically cross-linked recombinant *Hv*Pho1 using glutaraldehyde. After 30 s of incubation with glutaraldehyde (0.15% v/v) the cross-linking of the *Hv*Pho1 dimer was apparent by SDS-PAGE with no evidence of higher oligomerization states (Fig 2C). Thus, we considered if the high apparent molecular weight in SEC analysis could be due to the presence of other proteins in complex with Pho1.

We analyzed SEC fractions containing *Hv*Pho1 with antibodies targeted against branching enzyme IIb (*Hv*BeIIb), since these two proteins have been reported to have a functional interaction which could also be physical [10], but *Hv*BeIIb does not appear to co-elute with *Hv*Pho1 (Fig 2D). Fractions containing *Hv*BeIIb instead eluted at a position which corresponds to a dimer of 190 kDa. No changes in the elution profile of *Hv*Pho1 or *Hv*BeIIb occurred after ATP treatment of endosperm extracts either (Fig 2D). We also tested the SEC behavior of recombinant *Hv*Pho1 in mixtures with recombinant branching enzyme I (*Hv*BeI), branching enzyme IIa *(Hv*BeIIa) and *Hv*BeIIb. Each protein eluted at its expected elution volume, hence we could not find any interaction between these three branching enzymes and *Hv*Pho1 *in vitro*.

Pho1 contains a specific insertion that is not found in Pho2 enzymes. This insertion was named L78 in sweet potato *Ib*Pho1 based on its length. It was suggested that L78 forms a flexible loop in Pho1 enzymes [17,18]. However, the exact function of this insertion has not been elucidated. The L78 insertion within the GT35 domain of *Hv*Pho1 contains a large number of negatively charged amino acids. This chemical property could account for an enlarged hydrodynamic radius giving rise to the higher apparent molecular weight of 330 kDa for *Hv*Pho1.

For functional characterization of the significance of the L78 insertion, we produced a truncated version of *Hv*Pho1 that lacks the conserved L78 insertion (*Hv*Pho1ΔL78) as a soluble protein in *E*. *coli*. The apparent SEC molecular weight of *Hv*Pho1ΔL78 was very different from that of full-length *Hv*Pho1. In SEC, *Hv*Pho1ΔL78 eluted at a position corresponding to a molecular weight of approximately 180 kDa, roughly in agreement with the expected mass of a dimer. Analysis with DLS confirmed this change in apparent size (Fig 2E). We also assessed the enzymes’ affinity to large glucans *in vitro*. Both proteins, full-length *Hv*Pho1 and *Hv*Pho1ΔL78 could bind to amylopectin and starch (Fig 2F). However, binding of *Hv*Pho1ΔL78 to starch was slightly stronger than for the full-length protein, with Kd values of 0.015 mg∙ml^-1^ and 0.033 mg∙ml^-1^ respectively (Fig 2F).

### Structural analysis of *Hv*Pho1

**Fig 3:**
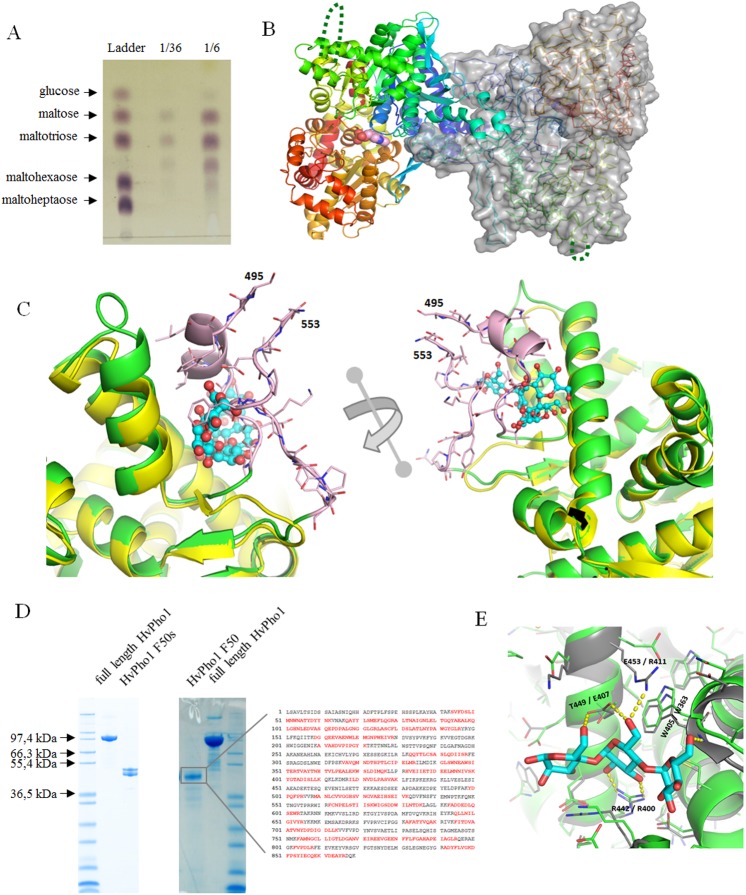
Crystal structure and modes of polysaccharide binding. (A) Thin Layer Chromatography (TLC) of a crystallization drop of *Hv*Pho1 containing 10 mM maltoheptaose. A ladder with 1 mM sugar markers is included and two dilutions from the crystallization drop were applied next to it. 1 μl of solution was loaded in each lane. (B) Overall structure of the *Hv*Pho1 dimer in the crystals. One monomer is shown as a semitransparent gray surface with the *α-*carbon trace as a stick model. The second monomer is shown as a cartoon model. Colors are in rainbow from dark blue in the N-terminus through light blue, green, yellow and orange to red at the C-terminus. The protein co-factor pyridoxal phosphate group is show as spheres (pink carbons) and lies at the interface between both, the N-terminal and C-terminal subdomains. The missing loops are indicated with dashed green lines. (C) Structural overlay of the native *Hv*Pho1 structure (green) and rabbit glycogen phosphorylase B (PDB-code 1P2B). 1P2B has maltoheptaose (shown as ball and sticks) bound in the glycogen storage site of glycogen phosphorylase B, although only maltopentaose has been explicitly modelled. 2 different angles are presented. Parts of *Hv*Pho1 corresponding to the L78 insert are colored pink with the last modeled residues indicated by sequence numbers. (D) Recombinant *Hv*Pho1, when stored at 15°C, degrades over time into specific degradation products called F50 and F50s. Left: initial degradation products after 1 week. Middle: a stable F50 band, indicated with a square, was apparent after 4 weeks incubation. It was excised from SDS-PAGE, gel eluted, trypsin digested and the resulting proteolytic fragments analyzed by MALDI-TOF. The sequence to the right shows full-length *Hv*Pho1. Labelled in red are MALDI-TOF recorded trypsin fragments (they do not cover the L78 insertion). (E) Superposition of *Hv*Pho1 (green carbon backbone) on the maltotriose binding site of *At*PHS2 (gray carbon backbone). The maltotriose from the *At*PHS2 crystal, not present in the *Hv*Pho1 structure, is shown with cyan carbons. For clarity, only side chains are shown except for W405/W363. Hydrogen bonds to the maltotriose in *At*PHS2 are highlighted with yellow dashed lines and the corresponding residues are labelled in the figure, first with the Pho1 residue numbering, then with the *At*PHS2 numbering.

We determined the crystal structures *of Hv*Pho1, the first crystal structure of a plastidic plant starch phosphorylase. A native structure of *Hv*Pho1 and two complexes with acarbose and maltotetraose were solved and refined to resolutions of 2.7 Å, 2.9 Å and 3.0 Å respectively. The native structure was obtained from a crystal formed from a drop in the absence of substrates, which was then mixed with another drop, to which 10 mM maltoheptaose had been added. The intention had been to obtain a crystal of the complex, but no maltooligosaccharide ligands were observed in the electron density. TLC analysis of a similar crystal-drop, where *Hv*Pho1 was co-crystallized with maltoheptaose, revealed that the maltoheptaose was hydrolyzed, mostly to maltose and maltotriose (Fig 3A). Accordingly no maltooligosaccharides were observed in the electron density of this *Hv*Pho1 crystal and we refer to it simply as the “native” crystal. Features from this native crystal are described unless noted otherwise.

Two almost identical *Hv*Pho1 monomers (R.M.S.D. = 0.19 Å), related by a quasi-crystallographic translation, are present in the asymmetric unit of the crystal. One of them had weak electron density for a citrate anion in the active site, likely a crystallization artifact as citrate was part of the mother liquor. Consequently, one citrate molecule was included in the final model. The structure is similar to those of the cytosolic phosphorylase of *A*. *thaliana* (*At*PHS2) [20], rabbit muscle phosphorylase B [21] and *E*. *coli* MalP [22], including the presence of a pyridoxal-5’-phosphate prosthetic group covalently bound to Lys814 and similar dimerization interfaces (Fig 3B). The Cα R.M.S.D. values are 0.76 Å relative to *At*PHS2, 1.45 Å relative to *Ec*MalP and 1.28 Å relative to rabbit muscle phosphorylase B (PDB codes 4bqe, 1e4o and 1p2b respectively).

Interpretable electron density extended from Ile69 to Lys495 and from Glu553 to Pro968 in all three crystals. Thus, our models include 12 amino acids at the N-terminus and 8 amino acids at the C-terminus of the L78 insertion. Residues 495 and 553 are contiguous to each other in space and would allow the L78 insertion to protrude away from the rest of the protein (Fig 3C). SDS-PAGE analysis of a dissolved *Hv*Pho1 protein crystal showed that no full-length protein is present after crystallization. Instead two well resolved bands at 50–55 kDa are seen (Fig 3D). The apparent sizes of both bands roughly match the mass of the N- and C-terminal halves of *Hv*Pho1 to each side of the L78 insertion. This finding suggests that the protein has undergone proteolysis in the crystallization drop. To test whether the protein degraded upon storage, we incubated *Hv*Pho1 after the last purification step (ion exchange) at 15°C for 4 weeks. Initially two fragments of approximately 50 kDa were formed, described in the literature as “F50s” [23]. These two fragments remained catalytically active as shown by HPAEC-PAD (S1 Fig), which indicates that they operate in one structural unit. This is also exemplified in the crystal structure of *Hv*Pho1 which lacks a large part of the L78 insertion. Gel retardation assays were used to investigate the affinity of this truncated version for starch. The migration patterns in gels containing starch indicate that binding of the F50s to starch was stronger than that of full length *Hv*Pho1 by an order of magnitude, with Kd for starch of 0.0025 mg·ml^-1^ for the F50s (Fig 2F), compared to 0.033mg·ml^-1^ for the full length protein and six-fold stronger than for *Hv*Pho1ΔL78 (0.015 mg·ml^-1^). Eventually, upon further incubation, only a stable single fragment remained (*Hv*Pho1F50, likely composed of two overlapping bands in the SDS-PAGE, Fig 3D). Trypsin digestion and MALDI-TOF analysis of this stable degradation product showed that it contained both the N- and C-terminal region of the protein (Fig 3D). No parts of the L78 insertions were found in either fragment. Hence, two fragments of the same size were stable proteolysis products which, upon formation, presumably promoted crystallization of *Hv*Pho1. It was also observed that crystallization was faster for *Hv*Pho1ΔL78, with crystals appearing after 2 days compared to 2 weeks for intact *Hv*Pho1.

A prominent feature of glycogen phosphorylases is the glycogen storage site [24]. An overlay on rabbit muscle glycogen phosphorylase b [24] suggests that *Hv*Pho1 does not contain a functional polysaccharide storage site (Fig 3C). The ordered part of the L78 insertion overlaps with the location where glycogen (or starch) binds to phosphorylase, in a position incompatible with maltooligosaccharide binding. This finding is consistent with previous experimental results suggesting that the L78 insertion provides a steric hindrance for large polysaccharides like starch or glycogen.

The *At*PHS2 structure revealed a maltotriose molecule bound in a surface site centered on threonine 449 (*Hv*Pho1 numbering), located adjacent to the glycogen storage site [20], which presumably also can contribute to affinity for glycogen and polysaccharides. We did not observe any bound ligands there. Structural comparison (Fig 3E) reveals that the area is likely not an α-glucan binding site in *Hv*Pho1. In particular, Glu407 (*A*. *thaliana* numbering), central to this binding in the *At*PHS2 structure via two hydrogen bonds, is substituted by Thr449 in *Hv*Pho1; while at the same time, Arg411, key to orienting Glu407 for binding, is substituted by Glu453 in *Hv*PHo1, which in turn adopts a significantly different conformation due to its interaction with a neighboring tryptophan. Thus, while no steric clashes are apparent, key features leading to glucan binding in this area in *At*PHS2 are missing in *Hv*Pho1, which should further contribute to the reduced the affinity of *Hv*Pho1 for polysaccharides. Notably, while residues equivalent to *At*PHS2’s Glu407 and Arg411 are present in MalP [22], an enzyme with low affinity for glycogen, a surface loop that contributes to binding this maltotriose is missing in MalP (comprising residues 256 to 261 in our *Hv*Pho1 structure).

**Fig 4:**
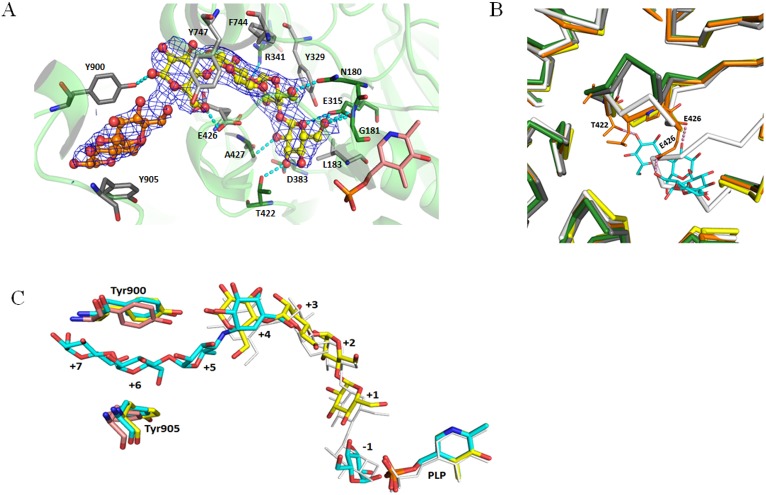
Structural details of the active site of *Hv*Pho1 and acceptor recognition. (A) Mode of binding of maltotetraose in the active site of *Hv*Pho1 (which is depicted as a semi-transparent ribbon). Maltotetraose is shown as ball and stick with yellow carbons and can also be seen in the same color in panel C. A modeled maltose is in ball and stick with orange carbons. Contacting amino acids are shown as stick models with green carbons, while those involved in stacking interactions with the glucose units are depicted with gray carbons. The pyridoxal phosphate is also depicted, with pink carbons, in the lower left corner. (B) Movement of a loop of *Hv*Pho1 in response to maltotetraose binding. Residues 422–427 of the *Hv*Pho1 complex with maltotetraose are highlighted with all atom sticks (orange carbons) and the maltotetraose is shown as sticks with cyan carbons. The thicker ribbons represent the α-carbon trace of *Hv*Pho1 bound to maltotetraose (orange), *Hv*Pho1 in the native structure (green), *Hv*Pho1 in complex with acarbose (gray), *Ec*MalP in complex with maltopentaose (yellow) and rabbit muscle glycogen phosphorylase (white). (C) Superposition of maltopentaose in the binding site of MalP (white, from PDB_code 1e4o), maltotetraose in *Hv*Pho1 and acarbose in *Hv*Pho1; plus PLP groups and selected details from the native *Hv*Pho1 structure. For clarity, only the mentioned groups, the pyridoxal-5’-phosphates (PLP) and, in the case of all three structures of *Hv*Pho1 reported here, Tyr900 and Tyr905 are depicted. Details from the maltotetraose complex are shown with yellow carbons, details from the acarbose complex with cyan carbons and details from the native structure with pink carbons. The four glucose units in the maltotetraose complex overlap well with the MalP structure for sub-sites +1 to +4.

In the crystal soaked with maltotetraose, one maltotetraose was found in each active site, with B factors around 90 Å^2^, in a position equivalent of that of the acceptor substrates in MalP structures [21,25]. The substrate hexose units occupy subsites +1 to +4, with the ~6 Å gap between the non-reducing end of the acceptor and the pyridoxal-5’-phosphate corresponding to the (unoccupied) subsite -1 (Fig 4A). The most notable difference to the native structure (and the acarbose soak) is a movement of a loop between Thr422 and Ala427 from an open to a closed conformation in response to acceptor binding. In particular, Thr422 through its side chain and Glu426 through the backbone carbonyl are hydrogen bonded to the acceptor in the maltotetraose soak (Fig 4B). This movement is equivalent to that observed in the *Ec*MalP structures in response to acceptor binding in the so-called MalP 380 loop [25]. The maltotetraose is largely held in place by stacking interactions, one for each glucose, plus one extra towards the center of the α-glucan arc with Glu426, which is observed in a double conformation loosely interacting with different glucoses (Fig 4A). Most hydrogen bonds and one apolar contact are made to the glucose in the non-reducing end. The glucose in subsite +3 has no short contacts to the protein, while the fourth glucose has two short contacts. This could explain the substrate preference of α-1,4-glucan phosphorylases for acceptor substrates larger than maltotriose. Further weak electron density is present adjacent to the maltotetraose, where maltose was modeled as described below.

The third crystal, soaked with acarbose and G1P, has no ordered electron density for either substrate in the active site. Some disordered electron density is present there, and two fragments in each monomer have been interpreted as glucose molecules in our final model, but they probably represent the average electron density of a mixture of many species, possibly including solvent water and glycerol. Correspondingly, the 422–427 loop is in the open conformation as in the native structure. Acarbose molecules were modeled, one for each monomer, in arc-shaped electron density going through a gate formed by Tyr900 and Tyr905 and adjacent to the active site (Fig 4C). This gate is already formed, albeit unoccupied, in the native structure. In the maltotetraose soak, this gate contains weak electron density in which maltoses, representing generic maltooligosaccharide fragments, were modeled with high B-factors of 140–160 Å^2^, one per monomer (Fig 4A). The acarbose molecules have their non-reducing ends overlapping with the position of the glucoses in subsite +4 in the maltotetraose soak. Together, these two crystal structures define a path for the acceptor maltooligosaccharide from subsite +1 to subsite +7 (Fig 4C), at which point the maltooligosaccharides would extend into the solvent. This particular gate is present in a similar conformation in *At*PHS2 [20] with a phenyalanine in the place of Tyr900, but it is absent in glycogen phosphorylases and MalP proteins, which have a deletion around the Tyr900 area and consequently a different α–carbon trace.

### De novo synthesis of α-glucans from G1P

**Fig 5:**
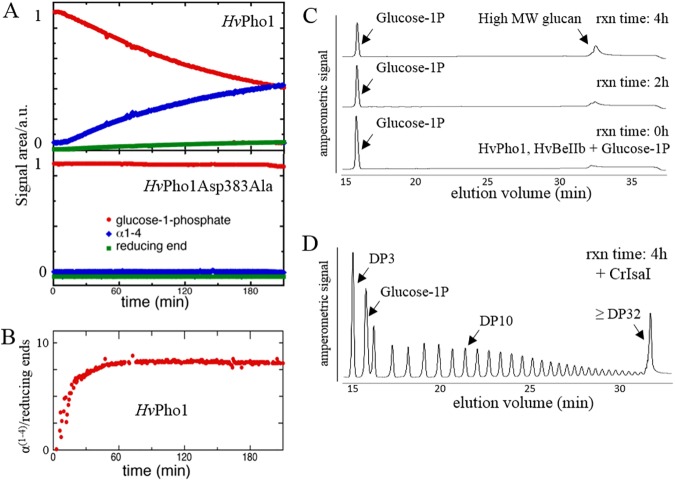
*De novo* production of α-1,4 glucans by *Hv*Pho1. (A) Proton NMR analysis of *de novo* synthesis of α-1,4 glucans by *Hv*Pho1. *Hv*Pho1 and *Hv*Pho1Asp383Ala (0,1 mg∙ml^-1^) were incubated with G1P (25 mM) as sole substrate. The figure shows production of 1,4 glycosidic bonds, usage of G1P and generation of reducing ends recorded over time by proton NMR spectroscopy. (B) Plot of the generation of 1,4 glycosidic linkages over the number of reducing ends indicating approximate lengths of glucans produced over time. (C) Recombinant *Hv*Pho1 (0.05 mg∙ml^-1^) and *Hv*BeIIa (0.05 mg∙ml^-1^) were incubated with G1P (50 mM). Samples were taken at the start of the reaction (0 h) after 2 h and after 4 h. (D) The last sample (4 h) from panel C was debranched using *Cr*Isa1 (0.01 mg∙ml^-1^) at 25°C overnight. Each sample was boiled, filtered and loaded on a CarboPac PA-100 ion-exchange column and analyzed with pulsed amperometric detection. The gradient used could not distinguish between species with more than 32 glucoses.

Although maltotetraose is commonly regarded as the smallest acceptor substrate for phosphorylase enzymes, roles in starch synthesis initiation have also been proposed for phosphorylase [6,10]. We assayed highly purified, recombinantly produced *Hv*PhoI with G1P *in vitro* and found it to efficiently catalyze the formation of long linear glucans (Fig 5A). To exclude the possibility that any external poly- or malto-oligosaccharide impurities were bound to *Hv*Pho1 we dialyzed the protein extensively after purification and pretreated all components with commercial pullulanase and α-glucosidase.

The reaction of a mixture of *Hv*Pho1 and G1P was followed via NMR over the course of 3.5 hours. In this reaction, glucose might be present in the final reaction buffer, particularly as an impurity of G1P. After a short lag phase, *Hv*Pho1 produced 1,4 linkages concomitantly with consumption of G1P (Fig 5A). *Hv*Pho1 catalyzed reaction maintains a steady α(1,4)/reducing end ratio just below 10 after an initiation phase of around 45 minutes. In a control reaction with an active site mutant of *Hv*Pho1 (Asp383Ala), no 1,4-glucan production occurred. *Hv*Pho1ΔL78 was also capable of *de novo* maltooligosaccharide synthesis as shown in S2 Fig.

To analyze whether the DP of *de novo* produced glucans is sufficient to act as substrates for barley branching enzyme, we incubated G1P together with recombinant *Hv*Pho1 and *Hv*BeIIa. Over the course of four hours, synthesis of high molecular weight glucans was observed (Fig 5C). Debranching of those high molecular weight glucans via recombinant isoamylase 1 from *Chlamydomonas reinhardtii* (*Cr*Isa1)[26] resulted in a very disperse pattern of branching ranging from DP3 to above DP32 (Fig 5D). Interestingly, the most abundant branch length was DP3 followed by DP4, along with a broad maximum at DP7 and DP8. The accumulation of maltotriose most likely reflects the preference of *Hv*Pho1 to act on substrates with a DP of 4 or larger.

### Possible synergistic role of *Hv*Pho1 and *Hv*BeIIa during endosperm development

It has been mentioned that *Hv*Pho1 was already detected at 0 DAF. To analyze whether any barley branching enzyme is present during the initial stages of barley endosperm development, endosperm protein extracts between 0 and 24 DAF were analyzed for the presence of *Hv*BEI, BeIIa and BeIIb via immunoblotting. Interestingly, *Hv*BeIIa was the only branching enzyme detectable already at 0 DAF (S3 Fig), while *Hv*BeIIb and *Hv*BeI are only present after 12 and 16 DAF, respectively [27]. Considering the synthesis of high molecular weight glucans by *Hv*Pho1 and *Hv*BeIIa *in vitro*, this is suggestive of the possibility for a synergistic role of these two activities in starch deposition.

## Discussion

### The L78 insertion affects the apparent oligomerization state, glucan binding and crystallizability of PhoI

The regulatory mechanisms of α-1,4-glucan phosphorylases may vary depending on the source of the enzyme [9,28,29] or on its multimeric state and association with other proteins [30]. Studies in maize suggest a more complex oligomeric arrangement. *Zm*Pho1 forms higher molecular weight protein complexes that depend on the phosphorylation state of the protein during maize endosperm development [31,32]. Most of the characterized α-1,4-glucan phosphorylases form dimers when analyzed in solution or via X-ray crystallography [25,29], including all known bacterial α-1,4-glucan phosphorylases and many plant α-1,4-glucan phosphorylases [25,29,33,34].

Thus, it was unexpected to find that *Hv*PhoI from plant extracts displays an apparent molecular mass of 330–350 kDa, corresponding to an apparent trimer with a monomer mass of ~105 kDa. The presence of a trimer in solution would be difficult to explain in a protein from a family known to form dimers and is at odds with our own cross-linking data. Regarding the possible occurrence of PhoI in heteromeric complexes, we could not detect the presence of any of the putative binding partners in the fractions showing phosphorylase activity and binding to anti-Pho1 antibodies. In addition, the fact that the same apparent molecular mass was measured for recombinantly expressed and purified *Hv*PhoI strongly argues against heteromeric complexes being responsible for the high apparent molecular mass.

**Fig 6:**
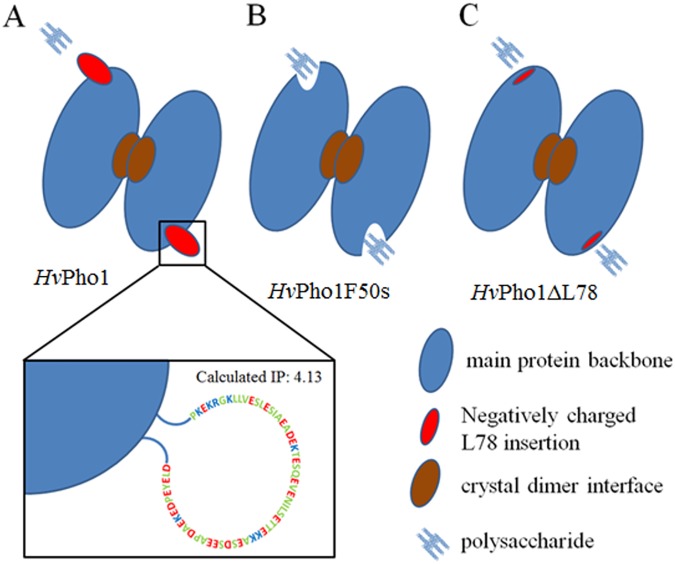
Model describing the effect of the L78 insertion on polysaccharide binding to *Hv*Pho1. (A) *Hv*Pho1 forms a homodimer in solution with an enlarged molecular size. The formation of the dimer is brought about by the crystallographic dimer interface. The flexible nature of the L78 insertion could block access of larger glucans to the protein´s surface. (B) The specific degradation products of *Hv*Pho1 are the F50s which probably lack L78. Our crystal structure does not contain the L78 insertion and might therefore represent the F50s rather than the full-length enzyme. The F50s provide better access to larger polysaccharides like starch or amylopectin. (C) *Hv*Pho1ΔL78 lacks the L78 insertion but it also lacks a break in the protein chain. Affinity of larger polysaccharides is similar to full-length *Hv*Pho1 as the main protein backbone is closed and restricts access to this area.

The most prominent feature distinguishing plastidial Pho1 from cytosolic Pho2 is the presence of the so-called L78 insertion between the two subdomains of *Hv*Pho1. We expressed *Hv*Pho1 recombinantly and purified a variant (*Hv*Pho1ΔL78) lacking this insertion. The resulting protein was catalytically active and exhibited an apparent molecular mass in solution of 180 kDa, which roughly corresponds to the expected mass for a dimer. The L78 insertion is, as shown in the crystal structures, far away from the dimerization interface. It is therefore concluded that *Hv*Pho1 is a homodimer in solution and that the high apparent molecular mass is an artifact induced by the presence of the L78 insert. The L78 sequence contains an unusually high proportion of negatively charged residues (Fig 6). This region can thus be expected to occupy a relatively large volume due to electrostatic repulsion. This extended volume would explain the higher apparent molecular mass as measured by SEC, as the dimer would be excluded from many spaces in the porous matrix, and as measured by DLS, as the increased hydrodynamic radius would slow down diffusion. This observation is compatible with the fact that crystallization of Pho1 only took place after cleavage of the L78 insert, and it was accelerated when *Hv*Pho1_ΔL78 was used directly for crystallization. The large volume occupied by the L78 insert would hamper the formation of a crystal matrix as this requires Pho1 dimers to be in close proximity to other dimers.

The L78 insert further proves to have an impact on the stability of *Hv*Pho1. Previous studies on sweet potato have shown that *Ib*Pho1 is cleaved into two fragments named F50s according to their approximate molecular weight of 50 kDa [23]. Native gel analyses with plant protein extract from Arabidopsis have identified up to five distinct phosphorolytic activities that were attributed to the two isoforms *At*PHS1 and *At*PHS2 [35]. Here, we have detected the formation of similar fragments in samples used for crystallization and we failed to detect any parts of the L78 insert after protein cleavage into these F50 fragments. It is thus possible that the L78 insert can also be cleaved *in vivo* in barley plastids. Whether this cleavage is mediated by an interaction with the 20S proteasome as identified in sweet potato by Lin et al. [23] remains unknown.

Removal of the L78 insert resulted in 2-fold increased affinity for glucans in the *Hv*Pho1_ΔL78 and 13-fold increased affinity in the presence of only the F50 fragments. The presence of the L78 insert thus seems to obstruct the binding to large, highly branched polysaccharides. Such an effect was also observed in sweet potato, where the presence of the L78 insert blocked the starch binding site in *Ib*Pho1 resulting in low affinity towards starch [36]. Such behavior is in agreement with conclusions drawn from enzymatic analysis on a chimeric enzyme composed of potato *St*Pho1 and *St*Pho2. In this enzyme chimera, the L78 insertion of *St*Pho1 including flanking sites was swapped with the corresponding sites in *St*Pho2 resulting in increased affinity for glycogen and starch substrates [37].

The significant modulation of hydrodynamic properties of *Hv*Pho1 by the L78 loop is consistent with an impact of the loop on substrate binding as detected in functional assays. The presence of the L78 insert could thus act as a regulatory element for Pho1 activity. Affinity and activity of Pho1 for large branched glucans can be increased by cleavage and even further by removal of the L78 insert as illustrated(Fig 6). Additional roles for the L78 insert could include the involvement in the formation of complexes with other proteins [32] which were found to be dependent of phosphorylation, or providing thermal stability [38].

### Temporal abundance of Pho1 in the developing barley grain

Our results show Pho1 to be present and active in the developing grain already at 0 DAF. Both the abundance of Pho1 and its activity increased greatly after 12 DAF, but while the abundance of Pho1 continued to increase from there until 24 DAF, the activity levels as measured by zymograms decreased steadily from 12 DAF. This is in agreement with transcriptional data presented in [11], although the latter increase in protein abundance could not be deduced from that data and the timing varies slightly. Thus, some sort of post-translational regulation may influence activity of Pho1 between 12 and 24 DAF. Redox controlled activity of starch active enzymes has been documented before [39–42] and is thus a plausible explanation for the observed decrease in specific activity of *Hv*Pho1. However, our experiments with different redox active agents did not indicate any alteration of Pho1 activity, while the activity of other starch active enzymes was clearly affected. These results indicate that post-translational modifications other than redox control or protein degradation are likely responsible for the observed decrease of *Hv*Pho1 activity. Such post-translational modifications could include cleavage of the L78 insert. Although this was not evident in our zymograms between 12 DAF and 24 DAF, a Pho1 species with reduced apparent mass was detected by zymograms and was especially prominent at 2 DAF. This species was associated with the insoluble endosperm fractions as opposed to the soluble fraction, a behavior compatible with the expected increase in glucan affinity resulting from removal of the L78 insert. Other modifications, including phosphorylation, were not studied in this work but are also conceivable.

### De novo synthesis of glucans by *Hv*Pho1

The smallest acceptor substrate for starch synthases is maltose [27,43]. There is currently no evidence that supports the presence of a glycogenin-like activity that could explain the initiation of starch biosynthesis in the plant cell by starch synthases. An enzymatic *de novo* synthesis of α-1,4 glucans in starch producing compartments like plastids could initiate starch biosynthesis. Earlier reports have shown that *Hv*Pho1 is able to produce α-1,4 glucans *de novo* only using G1P as substrate [44,45]. Since those experiments were done on protein preparations from plant extracts, this synthesis could be attributed to minute amounts of maltooligosacharide impurities in the protein preparations that could be sufficient in quantity to prime a seemingly “*de novo*” transfer reaction.

Our experiments show that *Hv*PhoI is able to synthetize linear α-glucans from G1P in the absence of any oligo- or polysaccharide initiators. Furthermore, the ΔL78 construct was also capable of catalizing a qualitatively equivalent reaction, although with different kinetics.

The most important finding is the nature of the products, linear α-1-4-glucans, potentially of sufficient length as to act as substrates for branching enzymes (Fig 5B shows average, not maximum chain lengths), at which point the conditions for starch synthesis initiation would be fulfilled. The observed synthesis could be explained by the presence of small amounts of glucan impurities, for example originating from the purification process. We took extensive measures with dialysis of all buffers and protein preparations in the presence of glucan degrading enzymes to try to ensure that this was not the case. The fact that the reaction of full length *Hv*Pho1 continued to proceed and create extra α-1-4 bonds after 60 minutes while consuming G1P argues against glucan impurities being the origin of this activity. By that time the average chain length of the glucans was stable and the continued reaction must be due to continued initiation events. It can be argued that putative glucan impurities present in small amounts would have been consumed by this point, thus continuous new initiation of glucan synthesis from G1P is the most likely hypothesis. Importantly, the kinetic profile obtained by in situ NMR spectroscopy exhibits a clear lag phase at the beginning of the reaction both for the wild type and L78 deletion mutant, indicative of polymerization only upon initiation in situ rather than polymerization from pre-existing carbohydrates. Finally, the observed initiation by *Hv*Pho1 is in agreement with recent reports in rice that α-1,4 glucans could be generated solely by *Os*Pho1 and G1P in combination with any of the three rice branching enzymes [10]. It was suggested that interaction of *Os*Pho1 with rice branching enzyme (*Os*BeI, BeIIa or BeIIb) initiates activity on G1P as sole substrate and that the products of this *de novo* synthesis of glucans were highly branched glucans. While our data suggest that G1P is the only molecule involved in this initiation reaction, we cannot rule out that molecules of glucose could act as the initial acceptor. It is possible that some glucose was present during the reaction, either as an impurity in G1P or as a product of hydrolysis of G1P. In any case, a reaction scheme with glucose as the initial acceptor would remain a biologically viable path to glycan synthesis initiation.

Initiation of starch synthesis, in particular amylopectin formation, requires cooperation of phosphorylase with branching enzymes. No physical complex formation between Pho1 and any of the branching enzymes could be detected *in vivo*, even after treatment with plant extract and ATP, or *in vitro* with recombinant proteins. A functional interaction without direct physical contact would also fulfill the role as an amylopectin initiator, and such an interaction has been proposed in rice [10], where *Os*Pho1 activity in the presence of BeI, BeIIa or BeIIb was sufficient to start the synthesis of branched glucans. The same observation is made here with recombinantly produced *Hv*Pho1 and *Hv*BEIIa. Co-incubation of both enzymes with G1P followed by debranching resulted in the formation of high molecular weight glucans qualitatively equivalent to those formed from glycogen or amylopectin after debranching, extending at least until a degree of polymerization of 32. Thus, the interaction between *Hv*Pho1 and *Hv*BeIIa is sufficient to produce large branched glucans akin to amylopectin with G1P as the only initial substrate. Since both enzymes are already present in grain extracts at 0 DAF, this interaction meets all the necessary conditions to act as an initiator of starch synthesis *in vivo*.

### Plausible role of *Hv*Pho1 in starch synthesis initiation

Taken together, the *in vivo* and *in vitro* data presented here suggest that *Hv*Pho1 has a role in starch biosynthesis in barley endosperm. During the initial stage of barley endosperm development at 0 DAF, *Hv*Pho1 is already present as an active enzyme. The early production of active *Hv*Pho1 coupled with its activity on G1P as the sole substrate suggests that Pho1 might have a role in the initiation of starch synthesis. A hypothetical three-step model for the involvement of Pho1 in starch biosynthesis in barley endosperm is presented as supplementary information (S1 File and S4 Fig).

Starch phosphorylase activity probably favors starch degradation under most physiological conditions, with an equilibrium constant close to 1 and prevalence of phosphate over G1P, which makes it a non-viable candidate for the bulk of starch synthesis. This predominant role in degradation is further highlighted by the fact that proteobacteria, which lack a glycogenin initiator for glycogen synthesis, can synthesize glycogen based on the glycogen synthase glgA alone or on the amylomaltase malQ alone, but not on the glycogen phosphorylase glgP alone [46,47]. Furthermore, a recent study in *A*. *thaliana* [48] clearly points to a net degradative role for Pho1 in planta.

Nonetheless, specific functions of starch phosphorylase in starch synthesis remain possible. *In silico* studies based exclusively on thermodynamic considerations prove that phosphorylases are capable of creating broad distributions of maltooligosaccharides, including small fractions much longer than the average [14] with entropy as a significant driving force for the process. During the first days after pollination, the action of adenosine diphosphate glucose pyrophosphatase (AGPPase) will transform much of the ADP-Glucose meant for starch synthesis into G1P [49], which can then fuel some degree of elongation. Recently, potato plastidial starch phosphorylase has been used for the *in vitro* synthesis of amylose in the presence of a source of G1P and 30 mM phosphate [50], conditions that might somewhat resemble the plastidic environment when AGPPase is active. The same study [50] reported that removal of a region which included the L78 insertion reduced the catalytic efficiency of the enzyme. Thus regions adjacent to the L78 insertion may be important for this activity. Our crystal structure suggests an involvement of the region immediately before the L78 insertion in the binding of maltooligosaccharides via a loop in very close proximity to the gate formed by Tyr900 and Tyr905, while the region immediately after the L78 insertion contributes to the placement of said loop.

It is worth to consider that insertion of branching points into maltooligosaccharides generated by phosphorylase would act as a non-reversible stop point for degradation. Pho1 would then be unable to degrade the chain past the branching point, but would remain capable of elongating it further to recreate the ideal thermodynamic chain length distribution from the branching point on. Thus Pho1, which by itself would only produce very short maltooligosaccharides under physiological conditions, can produce much longer polysaccharides in synergy with branching enzymes, which effectively modify the equilibrium conditions.

This is only one of many possible starch synthesis initiation mechanisms and the presence of starch in a Pho1 knockout mutant in rice [7] proves that it cannot be the only one at work. This initiation mechanism could work redundantly with alternative initiation mechanisms, for example making short maltooligosaccharides that would serve as substrates for SSIII and SSIV [51] which could then carry the bulk of the elongation. Alternatively, phosphorylase function in G1P polymerization may also be a secondary mechanism active only in certain conditions, for example to guarantee proper starch synthesis initiation in cold conditions [38].

Until now, no *HvPho1* barley knock-out mutants have been identified. Recently however, it was shown that a reduction of *Hv*Pho1 protein levels in barley grains to under 30% of their normal value did not lead to any visible starch phenotype [52]. Nevertheless, less than 30% *Hv*Pho1 activity may be sufficient to drive production of primer molecules acting as initiator-glucans. Rice knock out mutants lacking *Os*Pho1 have an altered starch structure and display a shrunken kernel phenotype [7]. An effect of lacking *At*PHS1 on starch production in Arabidopsis is only observed under certain environmental conditions [16,48].

A detailed study of the effect of a lack of *Hv*Pho1 in barley and analysis of the possible associated loss of glucan primer production in barley *Hv*Pho1 knock-out plants may potentially provide further insight into the role of Pho1 in plants.

## Experimental procedures

### Plant material and tissue preparation

Barley plants (*Hordeum vulgare* ‘QUENCH’) were cultivated under standard greenhouse conditions at 18°C with 16 h of light and a relative air humidity of 60%. Developing seeds were harvested from the middle region of the ear at 2 d intervals starting from anthesis until 24 DAF. Pericarp and endosperm tissue fractions were separated by hand dissection with a light microscope. The plant material was immediately cooled on dry ice and the frozen material was powdered using a mortar and pestle. Subsequently the powder was dissolved in extraction buffer (100 mM MOPS pH 7.4, 150 mM NaCl, 0.1% Triton X-100, 10% glycerol, 1 mM DTT, 5 mM EDTA, 1% polyvinylpyrrolidone, plant protease inhibitor (P9599 –Sigma Aldrich)) and processed with a glass homogenizer. The resulting plant protein extract was separated into buffer soluble and buffer insoluble protein extract by centrifugation at 22.000 *g* for 30 min at 4°C.

### Expression of recombinant proteins

Recombinant HvTrxh2 and HvNTR2 were produced in *E*. *coli* and purified as described previously [53]. For protein expression of *Hv*Pho1 (GenBank accession No. AK369633) and starch branching enzymes *Hv*BEI, *Hv*BeIIa and *Hv*BeIIb (accession Nos. AAP72268.1, AAC69753.1 and AAC69754.1 respectively), genes were C-terminally tagged with a TEV-His_6_ sequence (FPDIENLYFQGGKPIPNPLLGLDSTHHHHHH) and synthesized with codon usage optimized for expression in *E*. *coli* (DNA20^®^: www.DNA20.com). The signal peptide was excluded in all protein constructs mentioned in this manuscript. *Hv*Pho1 mutant proteins (*Hv*Pho1_Asp383Ala and *Hv*Pho1ΔL78) were produced via quick change mutagenesis using PfuUltraII PCR master mix (Agilent Technologies^®^: http://www.genomics.agilent.com). To introduce the Asp383Ala mutation the following two mutagenesis primers were used: Fw: CAG ATG AAT AAC ACG CAC CCG; Rev: CGG GTG CGT GTT ATT CAT CTG. To delete the L78 insertion the following primers were used: Fw: CAT GCG GAC CAC ACG ATT ATC CAA GAT GCG CAT ATC; Rev: CGC ATC TTG GAT AAT CGT GTG GTC CGC ATG GCG AAC. *E*. *coli* Tuner cells containing the plasmids were grown overnight at 16°C after induction with IPTG (1 mM) at OD_600_ = 0.6. Cells were harvested by centrifugation and lysed with a cell disrupter in the presence of DNAse (Sigma, D5025), lysozyme (Sigma, 62970) and Complete Protease Inhibitor EDTA-free (Roche: www.roche.co.uk). After centrifugation and filtration to remove debris, extracts were subjected to chromatography on a HisTrap chelating column (GE Healthcare Life Sciences: www.gelifesciences.com), TEV protease treatment and an additional HisTrap step. Samples were loaded onto HisTrap columns using the following buffer: 100 mM HEPES (pH 7.5), 500 mM NaCl, 2 mM DTT, Complete Protease Inhibitor, 60 mM imidazole. Column bound proteins were eluted using the same buffer containing 500 mM imidazole. The second HisTrap was run with 20 mM imidazole and the flow through was collected before concentration and application onto SEC via a HiLoad Superdex S-200 26/60 prep grade column (GE Healthcare Life Sciences). SEC running buffer was 25 mM HEPES (pH 7.5), 50 mM NaCl. Finally, an ion exchange chromatography step was done on a Resource Q 6 ml column after samples have been dialysed against 20 mM TrisHCl pH 7.0. For dynamic light scattering, 20 μg protein was filtered (0.1 μm membrane) then analyzed in a 12 μl quartz cuvette in a dynamic light scattering device (DynaPro, Wyatt Technology Corp.: www.wyatt.com). Measurements were taken every 10 s at 25°C. Data were analyzed with the Dynamics™ software package.

### Crystallisation of *Hv*Pho1

Selected *Hv*Pho1 fractions from SEC were pooled and concentrated to 10 mg·ml^-1^. Of this stock, 0.9 ml were mixed with 0.1 ml of 100 mM maltoheptaose (Sigma M8253) or 100 mM acarbose (Sigma A8980) resulting in protein stocks of 9 mg·ml^-1^ with 10 mM substrate or inhibitor respectively. These stocks and the native protein without added substrate were used to screen for crystallization conditions. Initial crystallization conditions were identified using Crystal Screen I from Hampton Research. After optimization, the best crystals were obtained by the sitting-drop method using Cryschem plates (Hampton Research) from drops containing 1 μl of protein stock and 1 μl of precipitant composed of 0.2 M ammonium acetate, 0.1 M sodium citrate pH 5.6 and 30% PEG 4000 stored at 288 K. The crystal that we call native was from such a drop, which was then mixed with a similar drop made with protein stock containing maltoheptaose. For the acarbose crystal, crystallization conditions were the same. 0.5 μl of 80 mM acarbose and 20 mM G1P were added to the drop prior to mounting and freezing. For the maltotetraose crystal, 3 μl of reservoir solution mixed 1:1 with 100 mM maltotetraose were added to the drop prior to mounting and freezing. Crystals typically grew to approximate dimensions of 200 x 200 x 120 μm over one month. The crystals were mounted in Mitegen loops and frozen by plunging in liquid nitrogen.

### Data collection, processing and structural refinement of *Hv*Pho1

Diffraction data was collected in ESRF beamlines ID23-2 (for the maltotetraose crystal) and ID29 (for the native and the acarbose crystals). Diffraction data was reduced with XDS [54] with 3% of reflections flagged for each test set. Data quality and final refinement statistics are reported in Table 1. The native structure was solved by molecular replacement with MOLREP [55] using a truncated version of human liver glycogen phosphorylase (PDB_ID: 2ZB2) [56] as the search model. This then served as the initial model for the complex structures. The asymmetric unit has two monomers of Pho1 related by a pseudocrystallographic translation, which resulted in higher than usual R factors during both data processing and refinement of all crystal forms. Refinement was done with REFMAC [57] employing local NCS and TLS anisotropy restraints. Manual model building and map inspection was made with COOT [58]. Figures were rendered with PYMOL (www.pymol.org). Structural superpositions were created with the secondary structure matching algorithm in COOT.

**Table 1:** Data collection and refinement statistics.

	native	Maltotetraose soak	Acarbose soak
Dataset statistics			
Space group	C_2_	C_2_	C_2_
Unit cell parameters (Å)	a = 229.2, b = 63.5, c = 148.8, β = 115.2°	a = 227.7, b = 63.3, c = 148.3, β = 114.7°	a = 230.3, b = 63.7, c = 149.3, β = 115.1°
Wavelength (Å)	0.954	0.873	0.954
Resolution (Å)	2.70 (2.90)	3.0 (3.2)	2.90 (3.10)
Total observations	240015	146805	140529
Unique reflections	53331	38803	43219
Completeness (%)	99.0	99.7	98.0
Redundancy	4.5	3.8	3.2
Rmerge^#^ (%)	14.3 (114.4)	10.9 (121.8)	13.7 (115.3)
CC_1/2_ from XDS (%)	99.6 (66.6)	n/a	99.5 (59.9)
I / σ(I)	8.8 (1.3)	10.1 (1.1)	7.6 (1.0)
Refinement statistics			
Resolution range (Å)	2.70 (2.77)	3.00 (3.08)	2.90 (2.98)
R_work_^+^ (%)	21.7 (25.7)	19.5 (24.3)	21.3 (49.6)
R_free_^§^ (%)	57.8 (48.8)	37.0 (42.7)	24.6 (53.1)
No. non-H protein atoms	13506	13484	13481
No. water atoms	148	47	56
No. ligand atoms	43	166	152
Model statistics			
R.m.s.d. bonds (Å)	0.005	0.006	0.007
R.m.s.d. angles (°)	0.97	1.16	1.11
Av. B factor (Å^2^)	63.3	90.2	76.7
Ramachandran plot^*^			
Most favoured region (%)	95.2 (1609)	95.0 (1593)	95.5 (1613)
Allowed region (%)	4.5 (77)	4.5 (77)	4.2 (72)
Outliers (%)	0.3 (5)	0.5 (8)	0.3 (5)
PDB accession code	5LR8	5LRA	5LRB

Crystallographic statistics for the native, maltotetraose bound and acarbose bound structures. Numbers in parenthesis are for the highest resolution shell except for the Ramachandran plot, there the number of bonds is indicated.

#Rmerge = Σ_hkl_Σ_i_ |I_i_(hkl)—<I(hkl)>| / Σ_hkl_Σ_i_ I_i_(hkl).

+Rwork = Σ_hkl_ ||F_obs_|—|F_calc_|| / Σ_hkl_ |F_obs_|.

§Rfree = Σ_hkl_ ||F_obs_|—|F_calc_|| / Σ_hkl_ |F_obs_| calculated using a random set containing 3% of the reflections that were not included throughout structure refinement.

### Native gels for enzyme activity

To analyze *Hv*Pho1 activity in barley endosperm (~100 mg) developing seeds were harvested from the middle region of the ear at 2 d intervals starting from anthesis until 24 DAF. After samples were processed (see plant material and tissue preparation above) the protein concentrations were measured using Bradford reagent with bovine gamma globulin as protein standard. Samples (15 μg total protein on each well to assure equal protein loading) were mixed with native sample buffer and loaded onto acrylamide gels containing 0.25% (w/v) glycogen. After electrophoresis gels were washed with 100 mM citrate-NaOH pH 6.5 and then incubated for overnight at 37°C in the same buffer with 20 mM G1P. Finally native gels were stained with 0.67% (w/v) I_2_ and 0.33% (w/v) KI. For the analysis of the influence of redox conditions, the plant extracts were incubated for 2 h at 37°C in the presence of 1 mM oxidized DTT, 1 mM reduced DTT or a system containing 2 μM *Hv*NTR2, 8 μM *Hv*Trxh2, 0.7 mM NADPH and 10 mM EDTA prior to electrophoresis.

### Chemical cross-linking of HvPho1

Recombinant *Hv*Pho1 (1 mg∙ml^-1^, 600 μl) in 20 mM HEPES buffer (pH 7.5) was treated with 30 μl of a 2.3% (v/v) freshly prepared solution of glutaraldehyde for 0.5, 1, 2, 5, 10 or 30 min at 37°C. The reaction was terminated by addition of 10 μl of 1 M Tris-HCl pH 8.0 to 100 μl of the mixture. Cross-linked proteins were solubilized by addition of 70 μl of NUPAGE sample buffer and heating for 5 min to 95°C. This was then loaded onto NUPAGE 4–20% SDS-PAGE gels and run at 200 V for 1.5 h.

### Binding of recombinant proteins to amylopectin

Amylopectin (maize) was washed with 100 mM PIPES (pH 6.8) at 4°C. Samples of *Hv*Pho1 were mixed to a final concentration of 20 μM with 20 mg starch/glucan in 500 μl of this buffer, shaken for 30 min on ice and then centrifuged at 22,000 *g* for 5 min. The pellet was washed extensively with phosphate-buffered saline then incubated at 100°C for 10 min in 500 μl SDS-PAGE loading buffer. Soluble and pellet fractions were analyzed on 4–12% SDS-PAGE gels. To analyze the affinity of *Hv*Pho1 for starch, the migration pattern of the protein was assessed in native gels lacking or containing various concentrations of starch (from 0.01% to 0.5%). Data (Rf values) were plotted against the starch concentration in the gels. The resultant affinity constant for half maximum binding was calculated using Sigma Plot.

### Immunoblot analysis

Purified recombinant *Hv*Pho1, *Hv*BE1, *Hv*BeIIa and *Hv*BeIIb were used to immunize rabbits (Genscript^R^, USA Inc. 860 Centennial Ave. Piscataway, NJ 08854 USA). Bleeds were taken every 7 days during the course of 4 weeks. The serum of the final bleed was used in immunological experiments. Primary antibodies were used at a final concentration of 1:250. Secondary antibodies (Cy5-conjugated anti rabbit monoclonal antibodies) were used at a final concentration of 1:5000. To obtain semi quantitative data from the immunoblots the freely available ImageJ software was used to quantify bands based on known recombinant protein standards.

### Preparation of reaction mixtures for real-time observation of phosphorylase activity

Proteins (*Hv*Pho1, *Hv*Pho1ΔL78 and *Hv*Pho1Asp383Ala) were produced in *E*. *coli* and purified to high homogeneity as described above. To ensure that no dextrin impurities are present in the reaction mix, the G1P stock (500 mM) was incubated overnight with α-amylase (Sigma A3403) and pullulanase (SigmaP2986). This mixture was then boiled at 100°C for 5 min and centrifuged at 22000 g to separate the proteins from the G1P solution. *Hv*Pho1 and *Hv*Pho1ΔL78 (5 ml of 50 μg∙ml^-1^) were dialyzed with 20 changes of dialysis buffer in 250 ml dialysis buffer (50 mM sodium citrate pH 6.5, 25 mM NaCl). The dialysis buffer was prepared according to the following procedure: 1 l of 250 mM sodium citrate, 125 mM NaCl pH 5.0 was incubated with 5 ml pullulanase (Sigma P2986) at 22°C for 1 h. After incubation, the pH was increased to pH 6.9 and 5 ml of α-amylase (Sigma A3403) were added and the mixture was incubated for an additional hour at 22°C. The pH of the buffer was then adjusted to pH 6.5 before it was diluted to 5 L and stored at 4°C. This dextrin free buffer was then used as dextrin free dialysis buffer.

### Real-time observation of phosphorylase activity by in situ NMR

Reaction mixtures were freshly prepared for the *in situ* observation of phosphoylase activity (see above). To 400 μl of the buffer, 50 μl ^2^H_2_O (Cambridge Isotope Laboratories, Andover, MA, USA), 50 μl 500 mM G1P stock solution and 50 μl enzyme stock solution (1 mg∙ml^-1^) were added. Resultant reaction mixtures thus contained 50 mM HEPES buffer pH 7.0, 50 mM G1P and 0.1 mg∙ml^-1^ enzyme. Freshly prepared reaction mixtures were directly transferred to a 5 mm NMR tube and acquisition of high-resolution ^1^H NMR spectra was started on a 800 MHz Bruker (Fällanden, Switzerland) Avance II spectrometer equipped with a TCI CryoProbe and an 18.7 T magnet (Oxford Magnet Technology, Oxford, UK) Spectra were acquired at 25°C as pseudo-2D spectra looping over a standard Bruker pulse sequence (zgesgp experiment) that uses excitation-sculpting for water suppression. A total of 512 ^1^H spectra were acquired for each sample in this manner, sampling 16384 complex data points during an acquisition time of 1.27 s and employing a recycle delay of 1 s. For each ^1^H spectrum, 16 transients were averaged over a time of 38 s. Resultant 2D time profiles of phosphorylase-catalyzed reaction progression were processed in Topspin 2.1 (Bruker) with extensive zero filling using an exponential window function with a line broadening of 1 Hz. A baseline correction in the anomeric signal region of the spectrum was applied. The α-anomeric signals for glucose-1-phopshate, α-1,4 glycosidic bonds and reducing end were integrated in Topspin. Subsequent to the time series, a highly-resolved ^1^H-^13^C HSQC spectrum was recorded to validate the formation of linear α-1,4 glucopyranosyl chains by the ^13^C chemical shift [59][60].

### Amperometric detection of products from reactions including *Hv*Pho1 and *Hv*BEIIa

Reactions were done in 250 μl including 5μl *Hv*Pho1 (from a 1.5 mg·ml^-1^ stock) and 5μl of *Hv*BEIIa (from a 4 mg·ml^-1^ stock). The concentration of G1P was 50 mM. The reaction buffer was 50 mM MOPS pH 6.5. The reactions were boiled after different incubation times, spun down and diluted 1:10 before analysis (10 μl injected per run) in a DIONEX ICS-3000 system with pulsed amperometric detection and a Carbopac PA-100 column. For analysis, buffer A was 70 mM KOH and buffer B was 70 mM KOH plus 500 mM KOAc. A linear gradient was run going from 100% buffer A at 0 minutes to 40% buffer A, 60% buffer B at 30 minutes, followed by 98% buffer B to 35 minutes and 100% buffer A to 38 minutes. Debranching of an aliquot from the reaction incubated for 4 h was performed by adding *Cr*ISA1 to a final concentration of 0.01 mg·ml^-1^ and incubation overnight at 25°C.

### Thin Layer Chromatography (TLC)

TLC was performed with a protocol adapted from [61] on a TLC Silica gel 60 plate (Merck), loading 1 μL of aqueous solution per lane. Running solvent was ACN:EtOAc:PrOH:H_2_O in 85:20:50:50 proportions. Development was done with a 5% H_2_SO_4_ and 0.5% α-naphtol solution in EtOH followed by charring in a hot plate at 250°C.

## Supporting information

S1 Fig Amperometric detection of activity from the *Hv*Pho1 F50 fragments. Reaction mixtures with 10 mM maltoheptaose, 10 mM glucose-1-phosphate and 0.1 mg·ml^-1^ of different enzyme preparations were incubated for 1 hour at 37°C. Reaction and analysis conditions were otherwise the same as indicated in the main text for the reactions including *Hv*BEIIa. The product pattern is qualitatively the same as for the WT and Δ78 constructs of Pho1.
(TIF)

S2 Fig Proton NMR analysis of *de novo* synthesis of α-1,4 glucans by *Hv*Pho1ΔL78. *Hv*Pho1ΔL78 (0,1 mg∙ml^-1^) was incubated with G1P (25 mM) as sole substrate. (A) The figure shows production of 1,4 glycosidic bonds, usage of G1P and generation of reducing ends recorded over time by proton NMR spectroscopy. (B) Plot of the generation of 1,4 glycosidic linkages over the number of reducing ends indicating approximate lengths of glucans produced over time.
(TIF)

S3 Fig Temporal profile of abundance of *Hv*BEIIa in endosperm. Immunological detection of *Hv*SSIIa in the soluble fraction of endosperm extracts of grains from 0 to 24 DAF as indicated above the lanes. The correct bands, marked with a blue ellipse, are identified with the help of a lane loaded with recombinantly produced *Hv*BEIIa (red arrow and “Recomb” label). No *Hv*BEIIa was detected in the insoluble fractions.
(TIF)

S4 Fig Proposed hypothetical steps of starch biosynthesis in barley endosperm. Shown is a simplified model of a barley endosperm plastid and the principal set of reactions that drive starch biosynthesis during endosperm development. (A) During the initial stage of endosperm development *Hv*AGPPase is highly active and drives the production of G1P from ADP-glucose. *Hv*Pho1 is present as active protein able to produce glucans *de novo* that act as substrates for branching enzyme *Hv*BeIIa, possibly in concert with G1P produced by phosphoglucomutase. (B) During the second step, the branched glucans, produced by *Hv*BeIIa and *Hv*Pho1 are substrates for starch synthases and debranching enzymes that produce crystalline precursors for starch and thus initiate starch biosynthesis. (C) The last step is grain filling in which starch granules grow to macroscopic structures. The photo inserts in each developmental step picture the approximate developmental stage of a grain. ADP, adenosine diphosphate; ADPglucose, adenosine diphosphate glucose; AGPase, adenosine diphosphate glucose pyrophosphorylase; AGPPase, adenosine diphosphate glucose pyrophosphatase; ATP, adenosine triphosphate; DBE, starch debranching enzyme; Glucose6-P, glucose-6-phosphate; Glucose1-P, glucose-1-phosphate; PGM, phosphoglucomutase; PPi, pyrophosphate; PPiase, pyrophosphatase; SBE, starch branching enzyme; SS, starch synthase.
(TIF)

S1 File Hypothetical three-step model for the involvement of Pho1 in starch biosynthesis in barley endosperm. Described is a hypothetical three-step model for the involvement of Pho1 in starch biosynthesis in barley endosperm.
(PDF)
