# Supplementary figures and images for: Functional and structural characterization of plastidic starch phosphorylase during barley endosperm development
Source: PLoS One. 2017 Apr 13;12(4):e0175488. doi: 10.1371/journal.pone.0175488 (PMC5391026; doi:10.1371/journal.pone.0175488)

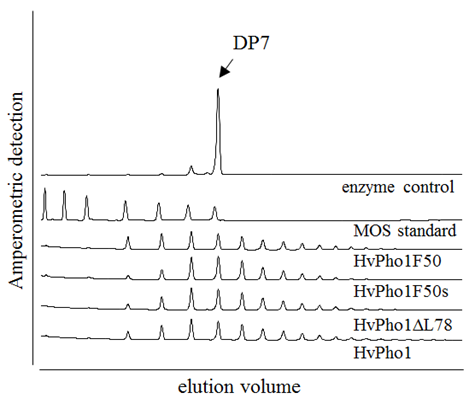

Supplement: S1 Fig — Reaction mixtures with 10 mM maltoheptaose, 10 mM glucose-1-phosphate and 0.1 mg·ml-1 of different enzyme preparations were incubated for 1 hour at 37°C. Reaction and analysis conditions were otherwise the same as indicated in the main text for the reactions including HvBEIIa. The product pattern is qualitatively the same as for the WT and Δ78 constructs of Pho1. (TIF) [file pone.0175488.s001.tif]

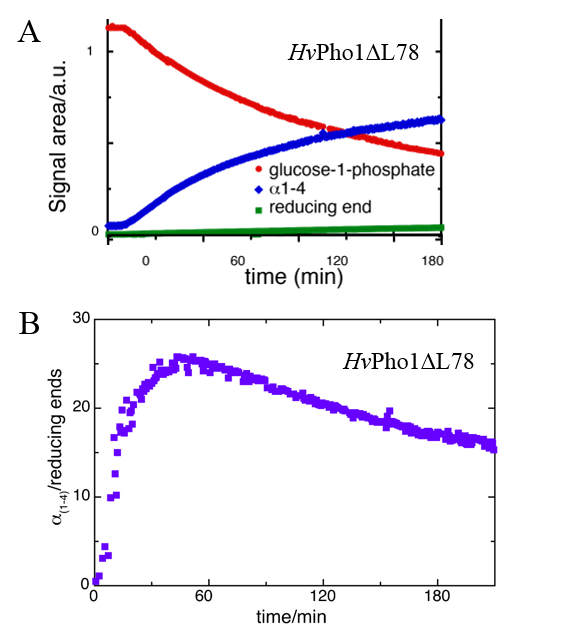

Supplement: S2 Fig — HvPho1ΔL78 (0,1 mg∙ml-1) was incubated with G1P (25 mM) as sole substrate. (A) The figure shows production of 1,4 glycosidic bonds, usage of G1P and generation of reducing ends recorded over time by proton NMR spectroscopy. (B) Plot of the generation of 1,4 glycosidic linkages over the number of reducing ends indicating approximate lengths of glucans produced over time. (TIF) [file pone.0175488.s002.tif]

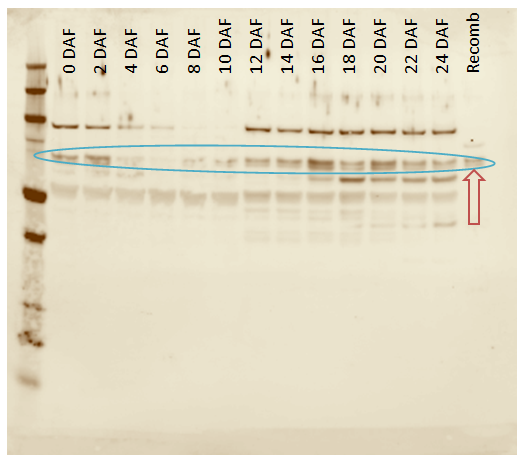

Supplement: S3 Fig — Immunological detection of HvSSIIa in the soluble fraction of endosperm extracts of grains from 0 to 24 DAF as indicated above the lanes. The correct bands, marked with a blue ellipse, are identified with the help of a lane loaded with recombinantly produced HvBEIIa (red arrow and “Recomb” label). No HvBEIIa was detected in the insoluble fractions. (TIF) [file pone.0175488.s003.tif]

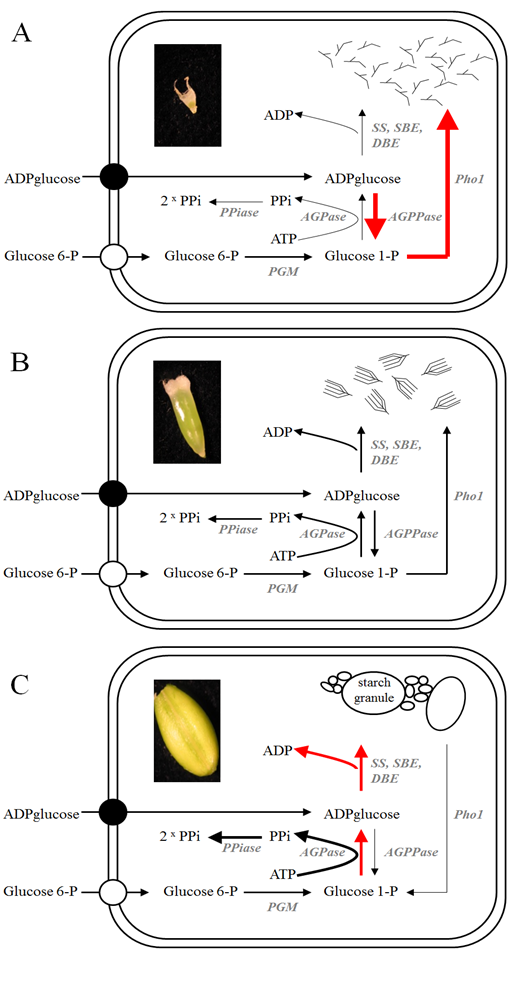

Supplement: S4 Fig — Shown is a simplified model of a barley endosperm plastid and the principal set of reactions that drive starch biosynthesis during endosperm development. (A) During the initial stage of endosperm development HvAGPPase is highly active and drives the production of G1P from ADP-glucose. HvPho1 is present as active protein able to produce glucans de novo that act as substrates for branching enzyme HvBeIIa, possibly in concert with G1P produced by phosphoglucomutase. (B) During the second step, the branched glucans, produced by HvBeIIa and HvPho1 are substrates for starch synthases and debranching enzymes that produce crystalline precursors for starch and thus initiate starch biosynthesis. (C) The last step is grain filling in which starch granules grow to macroscopic structures. The photo inserts in each developmental step picture the approximate developmental stage of a grain. ADP, adenosine diphosphate; ADPglucose, adenosine diphosphate glucose; AGPase, adenosine diphosphate glucose pyrophosphorylase; AGPPase, adenosine diphosphate glucose pyrophosphatase; ATP, adenosine triphosphate; DBE, starch debranching enzyme; Glucose6-P, glucose-6-phosphate; Glucose1-P, glucose-1-phosphate; PGM, phosphoglucomutase; PPi, pyrophosphate; PPiase, pyrophosphatase; SBE, starch branching enzyme; SS, starch synthase. (TIF) [file pone.0175488.s004.tif]
