# Supplementary material for: Functional and structural characterization of plastidic starch phosphorylase during barley endosperm development
Source: PLoS One. 2017 Apr 13;12(4):e0175488. doi: 10.1371/journal.pone.0175488 (PMC5391026; doi:10.1371/journal.pone.0175488)
Supplement: S1 File — Described is a hypothetical three-step model for the involvement of Pho1 in starch biosynthesis in barley endosperm. (PDF) [file pone.0175488.s005.pdf]

Hypothetical three-step model for the involvement of Pho1 in starch biosynthesis in barley endosperm (S4 Fig).

During the initial phase of barley endosperm development *Hv*AGPPase is present with high activity [1]. Using ADP-glucose as a substrate, *Hv*AGPPase produces G1P and thereby provides the substrate for the *de novo* production of linear α-1,4-glucans by *Hv*Pho1. Plastidial phosphoglucomutase might provide an alternative route for the production of plastidial G1P. *Hv*BeIIa, which is also already present at 0 DAF (Fig. S2), uses those linear α-1,4-glucans as substrates to produce simple branched glucans. Alternative *de novo* sources of linear glucans could be also present.

During the second stage of endosperm development (10 – 16 DAF), starch synthases could use the amylopectin like precursors produced by *Hv*Pho1 and *Hv*BeIIa as glucan initiators for the production of starch granules. At this stage starch production increases dramatically [2]. Activity levels of *Hv*AGPPase have been reduced several-fold at this point, thereby allowing accumulation of ADP-glucose via ADP-glucose pyrophosphorylase [1,2]. The role of *Hv*Pho1 might now be to assist the synthesis of starch by using the diminishing products of AGPPase, but with SSs already providing the main driving force.

During the third step of endosperm development after 18 DAF, the specific activity of *Hv*Pho1 decreases continuously. Starch elongation is now driven forward by the synthetic transfer of glucose residues by SSs producing substrates for branching and debranching enzymes. The predominant role of *Hv*Pho1 in this final stage of endosperm development could be glucan trimming to give rise to the ordered starch structure of the final starch granules [3] or could simply be the recycling of released linear maltooligosaccharides [4].

Supplementary references:

1. Rodríguez-López M, Baroja-Fernández E, Zandueta-Criado A, Pozueta-Romero J. Adenosine diphosphate glucose pyrophosphatase: a plastidial phosphodiesterase that prevents starch biosynthesis. Proceedings of the National Academy of Sciences. 2000;97: 8705–8710.

2. Radchuk VV, Borisjuk L, Sreenivasulu N, Merx K, Mock H-P, Rolletschek H, et al. Spatiotemporal profiling of starch biosynthesis and degradation in the developing barley grain. Plant physiology. 2009;150: 190–204.

3. Ball SG, Morell MK. From bacterial glycogen to starch: understanding the biogenesis of the plant starch granule. Annual review of plant biology. 2003;54: 207–233.

4. Watson K, McCleverty C, Geremia S, Cottaz S, Driguez H, Johnson L. Phosphorylase recognition and phosphorolysis of its oligosaccharide substrate: answers to a long outstanding question. The EMBO journal. 1999;18: 4619–4632.
